# A guide to ferroptosis in cancer

**DOI:** 10.1002/1878-0261.13649

**Published:** 2024-04-08

**Authors:** Fatma Isil Yapici, Christina M. Bebber, Silvia von Karstedt

**Affiliations:** ^1^ Department of Translational Genomics, Faculty of Medicine and University Hospital Cologne University of Cologne Germany; ^2^ CECAD Cluster of Excellence University of Cologne Germany; ^3^ Center for Molecular Medicine Cologne, Faculty of Medicine and University Hospital Cologne University of Cologne Germany

**Keywords:** cancer biology, cancer metabolism, cell death, ferroptosis, inflammation, iron

## Abstract

Ferroptosis is a newly identified iron‐dependent type of regulated cell death that can also be regarded as death caused by the specific collapse of the lipid antioxidant defence machinery. Ferroptosis has gained increasing attention as a potential therapeutic strategy for therapy‐resistant cancer types. However, many ferroptosis‐inducing small molecules do not reach the pharmacokinetic requirements for their effective clinical use yet. Nevertheless, their clinical optimization is under development. In this review, we summarize the current understanding of molecular pathways regulating ferroptosis, how cells protect themselves from the induction of ferroptotic cell death, and how a better understanding of cancer cell metabolism can represent vulnerabilities for ferroptosis‐based therapies. Lastly, we discuss the context‐dependent effect of ferroptosis on various cell types within the tumor microenvironment and address controversies on how tissue ferroptosis might impact systemic cancer immunity in a paracrine manner.

## Graphical abstract

Ferroptosis, an iron‐dependent form of regulated cell death, is gaining attention as a potential therapy for relapsed cancers. Despite challenges in pharmacokinetics, research is aiming for clinical optimization of ferroptosis inducers. Molecular pathways regulating lipid metabolism, radical trapping agents (RTA), iron metabolism, and systemic immunity in response to ferroptosis are discussed.

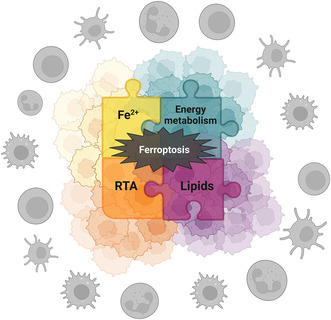

**Abbreviations**
4‐HNE: 4‐hydroxynonenal
7‐DHC: 7‐dehydrocholesterol
8‐OHdG: 8‐hydroxy‐2′‐deoxyguanosine
AA: arachidonic acid
ACSF2: acyl‐CoA synthetase family member 2
ACSL4: acyl‐coenzyme A synthetase long‐chain family member 4
AdA: adrenic acid
AGER: advanced glycosylation end‐product specific receptor
AKT: AKT Serine/Threonine Kinase 1
BH_4_
: tetrahydrobiopterin
BMDCs: bone marrow‐derived dendritic cells
BSO: buthionine sulfoximine
CAFs: cancer‐associated fibroblasts
CBS: cystathionine β‐synthase
CDO1: cysteine dioxygenase 1
c‐FOS: Fos proto‐oncogene
c‐JUN: Jun proto‐oncogene,
CoQH_2_
: ubiquinol
COXs: cyclooxygenases
CRC: colorectal cancer
CS: citrate synthase
D‐2‐HG: D‐2‐hydroxyglutarate
DAMPs: danger‐associated molecular patterns
DCs: dendritic cells
DGLA: di‐homo‐gamma‐linolenic acid
DHODH: dihydroorotate dehydrogenase
DLBCL: diffuse large B‐cell lymphoma
DMT1: divalent metal transporter 1
DPP4: dipeptidyl‐peptidase‐4
DPP4: dipeptidyl‐peptidase‐4
DTPs: drug‐tolerant persister cells
ECM: extracellular matrix
ER: endoplasmic reticulum
FAO: fatty acid oxidation
FATP2: fatty acid transport protein 2
Fe^+2^
: ferrous iron
Fe^+3^
: ferric iron
FH: fumarate hydratase
FINs: ferroptosis‐inducing agents
FPN1/SLC40A1: iron‐efflux pump ferroportin
FSP1: formerly known as AIFM2 oxidoreductase ferroptosis suppressor protein 1
FTH1: ferritin heavy chain
FTL: ferritin light chain
FXN: frataxin
GCH1: GTP cyclohydrolase 1
GCL: glutamate cysteine ligase
GCL γ: ‐glutamyl cysteine synthetase
GLS2: glutamine synthase 2
GPX4: glutathione peroxidase 4
GSH: glutathione
HMGB1: high‐mobility group protein B1
HO‐1: heme oxygenase‐1
IDH: isocitrate dehydrogenase
IKE: imidazole ketone erastin
iNOS: nitric oxide synthase
IREs: iron‐regulatory elements
IRP2: iron regulatory protein 2
IRPs: iron‐regulatory proteins
KEAP1: Kelch‐like ECH‐associated protein 1
LIP: labile iron pool
LOXs: lipoxygenases
LPCAT3: lysophosphatidylcholine acyltransferase 3
LRP8: lipoprotein receptor 8
MBOAT1/2: membrane bound O‐acyltransferase domain containing 1
MDSCs: myeloid‐derived suppressor cells
MEFs: mouse embryonic fibroblasts
MRP1: multidrug resistance protein 1
MUFAs: monounsaturated fatty acids
MYC: proto‐oncogene, basic helix–loop–helix (bHLH) transcription factor (MYC)
NCOA4: nuclear receptor co‐activator 4
NFS1: nitrogen fixation 1
NK: natural killer
NOXs: NADPH oxidases
NSCLC: non‐small cell lung cancer
NTBI: non‐transferrin bound iron
O·^−2^
: superoxides
O_2_
: oxygen
OXPHOS: oxidative phosphorylation
oxPLs: oxidized phospholipids
PARL: presenelin‐associated rhomboid‐like protein
PC: phosphatidylcholine
PDAC: pancreatic ductal adenocarcinoma
PDK4: pyruvate dehydrogenase kinase 4
PE: phosphatidylethanolamine
PGE2: prostaglandin E2
PI3K: phosphoinositide 3‐kinase
POR: cytochrome P450 oxidoreductase
PPRs: pattern recognition receptors
PROM2: prominin 2
PTEN: phosphatase and tensin homolog
PUFAs: polyunsaturated fatty acids
RB1: retinoblastoma‐associated protein 1
RTA: radical trapping agent
SAPE‐OOH: 1‐steaoryl‐2‐15‐HpETE‐sn‐glycero‐3phosphatidylethanolamine
SCLC: small cell lung cancer
SELENOP: selenoprotein P
SLC3A2/4F2: solute carrier family 3 member 2
SLC7A11/xCT: solute carrier family 7 member 11
STARD7: StAR‐related lipid transfer domain protein 7
STEAP3: six‐transmembrane epithelial antigen of prostate 3
STING: stimulator of interferon genes
TAMs: tumor‐associated macrophages
TCA: tricarboxylic acid
TfR1: transferrin receptor 1
TME: tumor microenvironment
T_reg_
: regulatory T cells
TRP14: thioredoxin‐related protein of 14 kDa
Txnrd1: thioredoxin reductase 1
VDAC2: mitochondrial voltage‐dependent anion channel 2
α‐KG: α‐ketoglutarate

## 1 Introduction

### 1.1 Hallmarks of ferroptosis

**Fig. 1:**
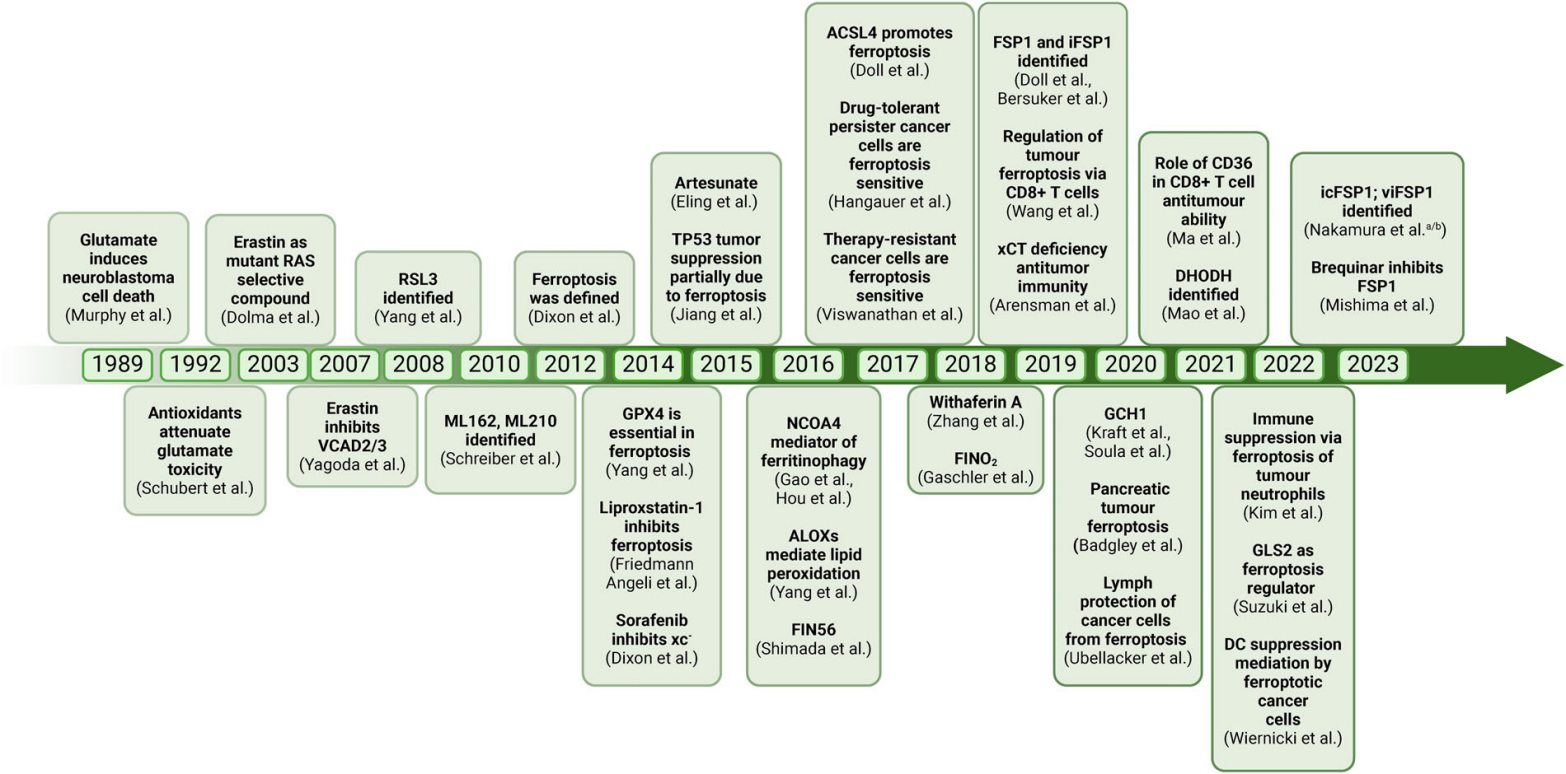
Timeline displaying cancer‐related ferroptosis landmark studies. A brief history of hallmark discoveries related to ferroptosis. Initial clues to ferroptosis via glutamate‐induced cell death were shown by Murphy et al., followed by the identification of pharmacological and molecular mediators that led to the eventual coining of the term ferroptosis by Dixon et al. in 2012. A figure has been created using Biorender.

Twenty years ago, in a quest to discover small molecules specifically targeting cancer cells, the Stockwell lab initiated a screening effort, ultimately leading to the discovery of a set of compounds that trigger a distinct form of oxidative cell death that did not involve the apoptosis or necroptosis molecular machinery [1]. Follow‐up studies showed that cells with Ras‐pathway activation underwent a selective type of cell death with necrosis‐like morphological features, including mitochondrial fragmentation, which was distinct from apoptosis, necrosis, or autophagy when exposed to erastin, a small molecule later identified to target solute carrier family 7 member 11 (SLC7A11 or xCT) [2]. Subsequent screening experiments showed that several iron‐chelating agents and lipophilic radical‐trapping antioxidants could inhibit this type of cell death [2]. Due to its dependence on iron, the term ‘ferroptosis’ was coined in 2012 by Dixon et al. [3] (Fig. 1). Ferroptotic cells are now known to present with loss of plasma membrane integrity, osmotic cytoplasmic swelling, and mitochondrial fragmentation and failure [4, 5]. Interestingly, in renal tubules and in cell culture, ferroptotic cell death propagates from cell to cell independently of cell rupture [5, 6], a feature that seems to be unique to ferroptotic cell death. Importantly, excessive lipid peroxidation is uniquely observed in cells undergoing ferroptosis [7, 8] marking it as one of the most defining hallmarks of ferroptotic cell death to date. Lastly, ferroptotic cells form small nanopores preceding cell rupture [9], suggesting a potential for the release of danger‐associated molecular patterns (DAMPs).

### 1.2 Iron: an enabling prerequisite for ferroptosis

**Fig. 2:**
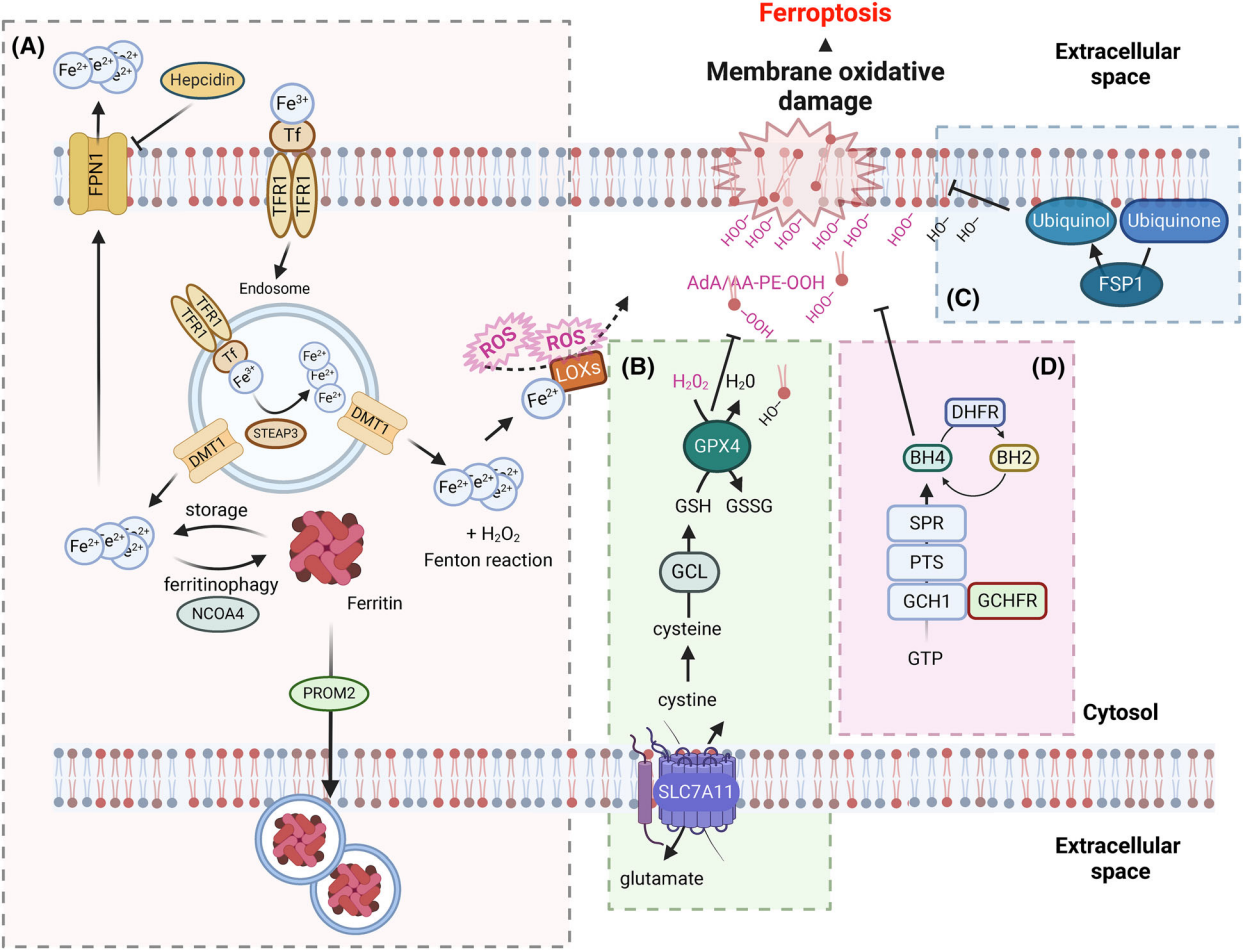
Schematic overview of ferroptosis regulation. Cellular metabolism modulates the sensitivity of cells to ferroptosis through the depicted signaling nodes such as iron metabolism (A) and antioxidant defense pathways (B, C, D). AdA/AA‐PE‐OOH, oxidized adrenic acid/arachidonic‐phosphatidylethanolamine; BH2, Dihydrobiopterin; BH4, Tetrahydrobiopterin; DHFR, Dyhydrofolate Reductase; DMT1, Divalent metal transporter 1; FPN1, Ferroportin 1; FSP1, Ferroptosis suppressor protein 1; GCH1, GTP Cyclohydrolase 1; GCHFR, GTP Cyclohydrolase 1 Feedback Regulator; GCL, Glutamate‐cysteine ligase; GPX4, Glutathione peroxidase 4; GSH, reduced glutathione; GSSG, oxidized glutathione; LOXs, Lipoxygenases; NCOA4, Nuclear receptor coactivator 4; PTS, 6‐Pyruvoyltetrahydropterin Synthase; PROM2, Prominin 2; ROS, Reactive oxygen species; SLC7A11, Solute carrier family 7 member 11; STEAP3, Six‐transmembrane epithelial antigen of prostate 3 metalloreductase; SPR, Sepiapterin Reductase; Tf, Transferrin; TFR1, Transferrin receptor 1. The figure has been created using Biorender.

The term ferroptosis was coined upon the finding that iron‐chelating small molecules inhibited it, and hence, this form of cell death was iron‐dependent [3]. Iron plays a vital role in ferroptosis, as it has the potential to generate highly reactive hydroxyl radicals through the Fenton reaction. This in turn initiates a chain reaction of lipid peroxidation [10]. In addition, iron functions as a cofactor of lipoxygenases (LOXs) and cytochrome P450 oxidoreductase (POR), both of which have been implicated in the direct generation of oxidized lipids [11]. Given that ferroptosis is dependent on iron, iron import and cellular availability can be considered as prerequisites for ferroptosis. Ferric iron (Fe^+3^), ferritin, and heme are the most common forms of iron absorbed throughout the body. Heme is a porphyrin‐class compound where a central iron atom is chelated by four nitrogen atoms and can be incorporated into proteins [12]. Macrophages constitutively phagocytose aged/dysfunctional erythrocytes, thereby providing an important route for systemic iron recycling. Upon lysis of erythrocytes, iron is released from hemoglobin by heme oxygenase‐1 (HO‐1) [13, 14]. Direct iron import into cells is mediated by the binding of transferrin‐bound ferric iron (Fe^+3^) to transferrin receptor 1 (TfR1), followed by endocytosis. Ferric iron (Fe^+3^) is then reduced to ferrous iron (Fe^+2^) by the six‐transmembrane epithelial antigen of prostate 3 (STEAP3). Fe^+2^ is then released into the cellular labile iron pool (LIP) by the divalent metal transporter 1 (DMT1), and TfR1 is recycled to the cell membrane [15]. TfR1 expression can be controlled via the five iron‐regulatory elements (IREs) that are located within its 3′ UTR [16]. Iron‐regulatory proteins (IRPs) can bind to IREs to upregulate TfR1 during iron‐deficient conditions [17]. The LIP is usually defined as the pool of redox‐active iron complexes, and since free iron is not stable, it is stored within the cell bound to ferritin, which includes two subunits: ferritin light chain (FTL) and ferritin heavy chain (FTH1). The nuclear receptor co‐activator 4 (NCOA4) can directly bind to FTH1 to degrade ferritin via lysosomes to increase free iron levels in the cell, a process known as ‘ferritinophagy’ [18, 19]. It can also be exported out of the cell by the iron‐efflux pump ferroportin (FPN1/SLC40A1) [20] or through prominin 2 (PROM2)‐mediated exosome‐dependent iron export with the formation of ferritin‐containing multivesicular bodies [21] (Fig. 2A). Diminished iron uptake and iron chelation block ferroptosis [2, 22], whereas removal of transferrin from serum prevents ferroptotic cell death [23]. While TfR1 upregulation increases ferroptosis sensitivity, silencing of iron regulatory protein 2 (IRP2) leads to a decrease in ferroptosis sensitivity [3, 22]. Conversely, suppression of TfR1 endocytosis through silencing of dynamins 1 and 2 is insufficient to suppress ferroptosis [24]. Iron metabolism is also tightly regulated by hepcidin, as it is involved in the internalization and degradation of FPN1 [25]. Hepcidin is an antimicrobial peptide that is synthesized and secreted by the liver and acts as a negative regulator of FPN1, causing iron overload in different tissues and thus contributing to ferroptosis [26] (Fig. 2A). Transferrin‐bound iron is the most common form under normal conditions, but in cases of iron overload, non‐transferrin‐bound iron (NTBI), including the LIP, also appears in the plasma [27]. NTBI comprises the non‐physiological, low‐molecular‐weight forms of iron that can be taken up by organs, leading to organ damage [28]. NTBI uptake also differs from that of transferrin‐bound iron [29]. As intracellular iron levels are closely controlled, the available iron pool can also be used for the synthesis of iron–sulfur clusters that usually function as cofactors in numerous mitochondrial processes [30] and also in non‐redox reactions [31]. Interestingly, defective synthesis of iron–sulfur clusters imposed by nitrogen fixation 1 (NFS1) inhibition/deletion or frataxin (FXN) suppression can activate an iron starvation response and sensitize cells to ferroptosis [32, 33]. Importantly, NFS1 expression is selected in lung cancer to protect against ferroptosis in highly oxygenated tissues [34]. Taken together, intracellular iron pools represent important prerequisites for many of the biochemical processes leading to lipid peroxidation.

### 1.3 GPX4‐dependent ferroptosis protection

Ferroptosis can be regarded as a cellular death caused by the collapse of lipid antioxidant defenses. As a result, lipoxygenase‐dependent and ‐independent peroxidation of arachidonic‐ and adrenic acid‐containing phosphatidylethanolamine (PE) and phosphatidylcholine (PC) membrane polyunsaturated fatty acids (PUFAs) has been shown to occur [7, 35]. Of note, these lipids are abundant within the endoplasmic reticulum (ER), and consequently, ER membranes present with lipid peroxidation in the early stages of ferroptosis [36]. Glutathione peroxidase 4 (GPX4) is a selenoprotein whose major function is to catalyze the reduction and detoxification of these phospholipid hydroperoxides and thereby protect from ferroptosis [4, 37, 38]. GPX4 exists in three different isoforms: cytosolic GPX4, which is the main isoform expressed; mitochondrial GPX4 (mGPX4) [39]; and nuclear GPX4 (nGPX4) [40]. However, only cytosolic GPX4 is an essential gene, as Gpx4‐deficient mice did not survive past midgestation [41] while mGPX4‐deficient mice were viable but presented with male infertility [42]. Importantly, the selenocysteine incorporated within the active pocket of GPX4 is required for full activity. Mice engineered to instead express a GPX4 variant with regular cysteine instead of selenocysteine suffer from excessive ferroptosis in interneurons, resulting in seizures [43]. To circumvent the problem of embryonic lethality, an inducible mouse model was generated, which showed that upon whole‐body induction of Gpx4 knockout, mice rapidly succumbed to lethal renal failure caused by excessive ferroptosis [4, 38]. In addition to selenocysteine, GPX4 activity depends upon its co‐factor and substrate glutathione (GSH) as an electron donor [44]. Therefore, a limiting supply of GSH equally results in ferroptosis.

GSH is a tripeptide synthesized from cysteine, glutamate, and glycine. Cells take up cystine via the cystine/glutamate antiporter (system xc^−^) that consists of two subunits: solute carrier family 3 member 2 (SLC3A2 or 4F2) and SLC7A11/xCT [3]. Once inside the cell, cystine is reduced to cysteine via NADPH consumption by the cystine reductases thioredoxin‐related protein of 14 kDa (TRP14) or thioredoxin reductase *1* (Txnrd1) [45, 46]. Cysteine is combined with glutamate by the enzyme glutamate cysteine ligase (GCL) to form γ‐glutamylcysteine, and as a last step, glycine is added to γ‐glutamylcysteine to form GSH by GSH synthase [47]. Inhibition of the xCT subunit of the cystine/glutamate antiporter by erastin was shown to result in ER stress and ferroptosis [48]. Of note, while xCT‐deficient mice are viable and fertile, xCT‐deficient mouse embryonic fibroblasts (MEFs) in culture rapidly die due to a lack of intracellular cysteine [49]. These data suggest that *in vivo*, transsulfuration pathway‐derived cysteine may compensate for the loss of xCT, while this does not seem to work in MEFs in cell culture. A possible underlying reason for this may be the fact that the *in vivo* redox environment might be more reducing, leading to a different cysteine/cystine ratio allowing for excess cysteine availability and uptake, which might compensate for the cell culture phenotype of xCT‐deficient cells. Moreover, prolonged xCT inhibition using erastin upregulated the transsulfuration pathway enzyme cystathionine β‐synthase (CBS) and increased ferroptosis resistance [50]. Similarly, directly impinging on the synthesis of GSH by inhibiting γ‐glutamyl cysteine synthetase (GCL) using buthionine sulfoximine (BSO) induces ferroptosis [37] (Fig. 2B). Sensitization to ferroptosis can also occur through the depletion of GSH via alternative mechanisms: multidrug resistance protein 1 (MRP1) causes a glutathione efflux, leading to ferroptosis sensitization [51] and cysteine dioxygenase 1 (CDO1) increases the sensitivity to ferroptosis by depleting cysteine and consequently glutathione levels [52]. While ER membranes have been identified as sites of ferroptosis‐associated lipid peroxidation, peroxidized membrane lipids and GSH depletion were also detected in mitochondria upon induction of ferroptosis [53]. Interestingly, a metabolism‐focused CRISPR‐Cas9 screen identified SLC25A39 as a major mitochondrial GSH importer [54] that protects from RSL3‐induced ferroptosis [55]. Apart from modulating its activity, two recent studies uncovered that GPX4 translation is a specific targetable vulnerability. Depletion of the selenoprotein P (SELENOP) receptor and low‐density lipoprotein receptor 8 (LRP8) sensitized cells to ferroptosis due to intracellular depletion of selenium/selenocysteine and ensuing stalling of GPX4 translation [56, 57]. Taken together, the currently known main arm of cellular ferroptosis protection revolves around GPX4 regulation of expression and activity through GSH synthesis, recovery, and localization (Fig. 2B).

### 1.4 Ferroptosis protection via the synthesis of endogenous radical‐trapping agents (RTAs)

A series of recent studies have revealed another concept by which cells protect themselves from ferroptotic cell death. First, the NADPH oxidoreductase ferroptosis suppressor protein 1 (FSP1, formerly known as AIFM2) was identified to potently protect cells from ferroptosis via the generation of ubiquinol (CoQH_2_) from coenzyme Q10 (CoQ10) [58, 59] (Fig. 2C). Myristoylation of FSP1 in its N‐terminus was required for plasma membrane recruitment, to increase local amounts of ubiquinol, which acts as a lipophilic radical trapping agent (RTA) [59]. The recently resolved crystal structure of FSP1 identified a much higher affinity for NADPH than for NADH, indicating a central role for NADPH as an electron donor within FSP1 [60]. Interestingly, cellular levels of NADPH correlate with ferroptosis resistance across human cell lines [61], which may be attributed to FSP1 activity (Fig. 2C). Surprisingly, plasma membrane recruitment of FSP1 was required for ferroptosis protection despite its substrate, CoQ10, being synthesized at the inner mitochondrial membrane, and it was not clear how CoQ10 might shuttle to the plasma membrane. Solving this conundrum, it was recently shown that the inner mitochondrial membrane protease Presenelin‐associated rhomboid‐like protein (PARL) cleaves StAR‐related lipid transfer domain protein 7 (STARD7) to generate a cytosolic form responsible for transporting CoQ10 to the plasma membrane, thereby protecting from ferroptosis [62]. While dihydroorotate dehydrogenase (DHODH) was described as a mitochondrial suppressor of ferroptosis through a similar RTA‐generating function in the inner mitochondrial membrane [63], this finding has recently been put in question. Evidence was put forward showing that brequinar – the DHODH inhibitor used in the original study – also effectively inhibits FSP1, leading to ferroptosis sensitization in DHODH‐deficient cells as well as in those that express the purported target, DHODH [64, 65]. Apart from CoQ10, FSP1 was recently shown to reduce vitamin K to its hydroquinone form, which also acts as a potent RTA protecting from ferroptosis [66]. GTP cyclohydrolase 1 (GCH1) was recently reported as a critical regulator of ferroptosis through its involvement in tetrahydrobiopterin (BH_4_) synthesis, a potent RTA that, in addition, can control the levels of CoQ10 as well as lipid remodeling [55, 67] (Fig. 2D). While this arm of ferroptosis sensitization is often branded as ‘GPX4‐independent’, this terminology has to be viewed critically given the fact that FSP1‐deficient cells do not spontaneously undergo ferroptosis but require either deletion or inhibition of GPX4 [58]. Moreover, FSP1‐deficient mice, unlike GPX4‐deficient mice, are viable and fertile [68]. Besides their important anti‐ferroptosis function as GPX4 co‐factors, cysteine and GSH can protect cells from ferroptosis independently of GPX4 by supplying sulfur for the formation of hydropersulfides, which have recently been described to counteract lipid peroxidation by terminating radical chain reactions [69]. Taken together, synthesizing endogenous RTAs is a potent strategy by which cells protect themselves from ferroptotic cell death induced by the collapse of lipid ROS detoxification.

### 1.5 Metabolic checkpoints of ferroptosis

#### 1.5.1 Lipid metabolism and turnover as a regulator of ferroptosis

PUFAs are specifically sensitive to peroxidation since their methylene double bonds contain highly reactive hydrogen atoms. Arachidonic acid (AA) and adrenic acid (AdA)‐containing PE‐phospholipids in the cell membrane, as well as ether‐linked PUFA‐containing phospholipids, which are synthesized in the ER, are predominantly peroxidized during ferroptosis [7]. High levels of ether lipid synthesis were separately shown to sensitize cells to ferroptosis in states of lipid plasticity [70]. The incorporation of these PUFAs into phospholipids is facilitated by acyl‐coenzyme A synthetase long‐chain family member 4 (ACSL4) [71] and lysophosphatidylcholine acyltransferase 3 (LPCAT3) [72], both of which are essential for a ferroptosis‐sensitive lipidome. ACSL4 preferentially acylates AA and AdA, which are then incorporated into phospholipids by LPCAT3, generating the target lipid pool sensitive to peroxidation during ferroptosis [73]. Moreover, feeding cells with exogenous AA sensitized them to ferroptosis [74]. Phospholipases A_2_ cleave phospholipids and have ferroptosis‐protective functions by either shaping the lipidome to contain more ferroptosis‐prone phosphatidylethanolamine phospholipids [75] or by cleaving peroxidized PUFAs at the sn2 position of phospholipids, thereby limiting chain propagation within cellular membranes [76, 77].

Whether ether‐linked PUFA‐containing phospholipids play a ferroptosis‐promoting or ‐preventing role is still under debate. A recent study showed that feeding human cancer cells the exogenous PUFA di‐homo‐gamma‐linolenic acid (DGLA) induces ferroptosis, which can be rescued either by treatment with ferropstatin‐1 or exogenous PUFA‐ether phospholipids, indicating a ferroptosis protective role for PUFA‐ether phospholipids [78]. However, Zou et al. [70] showed that endogenous PUFA‐ether phospholipid synthesized in peroxisomes promotes ferroptosis sensitivity and that knocking out genes involved in PUFA‐ether phospholipid synthesis renders cells more resistant to ferroptosis. In support of this, suppression of ether lipid synthesis rendered ferroptosis‐sensitive small‐cell lung cancer resistant [79].

Monounsaturated fatty acids (MUFAs) are less prone to lipid peroxidation, and the ratio of MUFAs to PUFAs therefore determines the speed at which lipid peroxidation propagates within membranes. Feeding cells with exogenous MUFAs such as oleic acid reduces ferroptosis sensitivity by increasing the MUFA/PUFA ratio and thereby impairing lipid peroxidation in membranes *in vitro* as well as *in vivo* [78, 80, 81]. The incorporation of MUFAs into membranes is facilitated by ACSL3. High expression of ACSL3 correlates with increased metastatic potential as well as ferroptosis resistance in some cancer cells [80, 81].

**Fig. 3:**
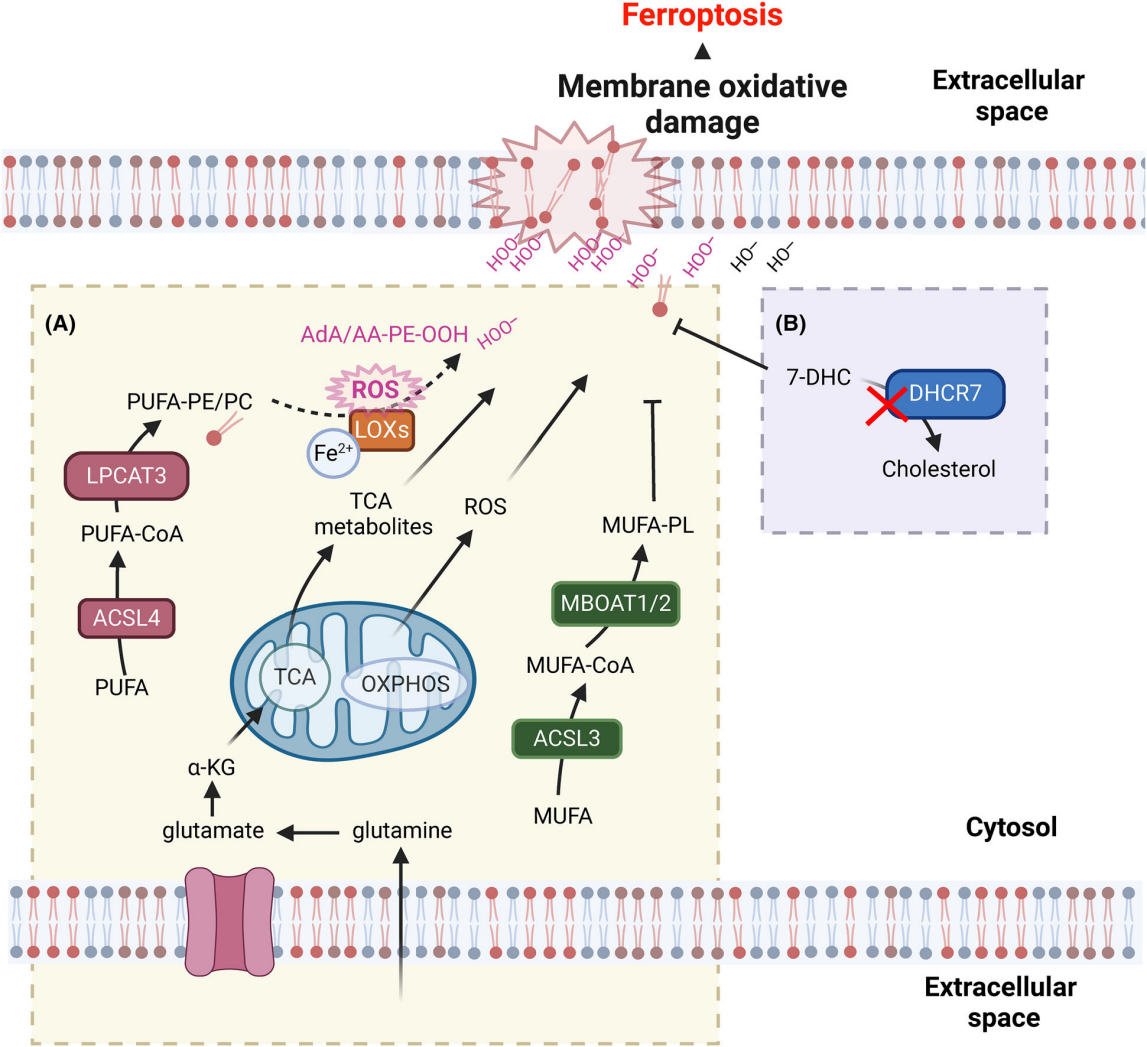
Metabolic regulators of ferroptosis. A and B, Ferroptosis sensitivity depends on lipid metabolism. 7‐DHC, 7‐Dehydrocholesterol; ACSL3, Acyl‐CoA synthetase long chain family member 3; ACSL4, Acyl‐CoA synthetase long chain family member 4; BH2, Dihydrobiopterin; BH4, Tetrahydrobiopterin; DHCR7, 7‐Dehydrocholesterol reductase; DHFR, Dihydrofolate reductase; GCH1, GTP cyclohydrolase 1; GCHFR, GTP cyclohydrolase 1 feedback regulatory protein; GTP, Guanosine triphosphate; LPCAT3, Lysophosphatidylcholine acyltransferase 3; MBOAT1/2 Membrane bound O‐acyltransferase domain containing 1/2; MUFA, Monounsaturated fatty acids; MUFA‐CoA, Monounsaturated fatty acid‐coenzyme A; MUFA‐PL, Monounsaturated fatty acid phospholipid; OXPHOS, Oxidative phosphorylation; PTS, 6‐Pyruvoyltetrahydropterin Synthase; PUFA, Polyunsaturated fatty acids; PUFA‐CoA, Polyunsaturated fatty acid‐coenzyme A; PUFA‐PE/PC Polyunsaturated fatty acid‐phosphatidylethanolamine/phosphatidylcholine; ROS, Reactive oxygen species; SPR, Spiapterin reductase; TCA, Tricarboxylic acid cycle; α‐KG, Ketoglutaric acid. The figure has been created using Biorender.

Membrane‐bound O‐acyltransferase domains containing 1 (MBOAT1/2) are MUFA‐specific lysophospholipid acyltransferases that remodel the lipidome to contain more MUFA‐phospholipids and thereby become more ferroptosis resistant (Fig. 3A). MBOAT1 and MBOAT2 are transcriptionally regulated by sex hormones. A combined estrogen receptor and androgen receptor antagonist treatment sensitized breast cancer to ferroptosis induction [82]. Moreover, a recent study describes that levels of MBOAT1 are decreased upon cell cycle arrest, resulting in increased ferroptosis sensitivity [83]. Another important determining factor for the regulation of ferroptosis is dietary lipid intake. A recent study found that feeding oleic acid to *Caenorhabditis elegans* and mice protects against iron‐overload toxicity by decreasing the amount of PUFA‐phospholipids and ether‐linked phospholipids [84]. Recently, two independent studies identified that 7‐dehydrocholesterol (7‐DHC) can restrict excessive lipid peroxidation during ferroptosis by trapping peroxyl radicals, thereby shielding phospholipids from autoxidation [85, 86] (Fig. 3B). Both studies identify a new protective role for B‐ring unsaturated sterols against phospholipid peroxidation and ferroptosis and suggest that truncation of phospholipids may explain nanopores observed during ferroptosis. Taken together, the PUFA‐ and MUFA‐containing lipid composition of cellular membranes is a strong determinant of ferroptosis sensitivity, which is subject to strict metabolic regulation and plasticity in cancer.

#### 1.5.2 Non‐lipid metabolism as a regulator of ferroptosis

Ensuing ferroptosis is currently defined by a set of chemical reactions (the Fenton reaction, a lipid peroxidation chain reaction) kicked off by imbalanced redox homeostasis. Therefore, pathways regulating the antioxidant redox capacity of a cell will affect ferroptosis induction and sensitivity. Apart from potent induction of ferroptosis through inhibition of cystine import (e.g., caused by erastin treatment), biosynthesis of cysteine from methionine via the transsulfuration pathway can serve as an alternative source of cysteine in cells refractory to this regime. Indeed, cysteine and methionine restriction in diet sensitizes glioma to ferroptosis *in vivo*, suggesting impinging on this pathway could represent a therapeutic vulnerability in this cancer context [87].

Mitochondria play a central role in cellular metabolism and generate the majority of cellular ROS as a by‐product of oxidative phosphorylation (OXPHOS) in the electron transport chain located within the inner mitochondrial membrane. During OXPHOS, electrons derived from NADH are transmitted through redox reduction reactions to the terminal electron acceptor oxygen (O_2_), which is reduced to H_2_O. Yet, a small proportion of electrons leak out and react with O_2_, forming highly reactive superoxides (O^·−2^). NADH is one of the major products of the tricarboxylic acid (TCA) cycle within the mitochondrial lumen, driven by metabolites such as acetyl‐CoA derived from glycolysis and catabolic fatty acid oxidation (ß‐oxidation). When ferroptosis is induced by cysteine starvation or erastin treatment, activity of the mitochondrial TCA is required for cells to undergo ferroptosis. Feeding cells α‐ketoglutarate (α‐KG) restores their potential to undergo ferroptosis, clearly demonstrating a ferroptosis‐promoting role for mitochondria [23]. Despite this, mitochondria are not the main site of lipid peroxidation [36], but TCA intermediates and enzymes influence ferroptosis sensitivity. Glutamine plays an important role in ferroptosis, and glutamine synthase 2 (GLS2), a key regulator of glutaminolysis, has been shown to promote ferroptosis [88]. GLS2 contributes to the degradation of glutamine, providing α‐KG for the TCA cycle. Interestingly, GLS2 has been described to have a tumor‐suppressive function in hepatocellular carcinoma [88]. GLS2 overexpression impaired tumor growth, which could be abolished by Ferrostatin‐1 co‐treatment, indicating that its tumor‐suppressive function is partially mediated by ferroptosis in hepatocellular carcinoma [88]. TCA cycle metabolites like α‐KG and its downstream products, such as succinic acid and fumaric acid, enhance ferroptosis induced by cysteine depletion [89].

Citrate synthase (CS) regulates fatty acid synthesis, while acyl‐CoA synthetase family member 2 (ACSF2) modulates fatty acid activation, serving as specific lipid precursors in fatty acid oxidation (FAO) [3]. Additionally, pyruvate dehydrogenase kinase 4 (PDK4) in the mitochondrial inner membrane has been identified as a top metabolic enzyme leading to ferroptosis resistance in a siRNA screen. PDK4 inhibits ferroptosis by suppressing pyruvate oxidation and fatty acid synthesis, facilitating lipid peroxidation‐dependent ferroptotic death [90]. Another important TCA enzyme is isocitrate dehydrogenase (IDH). Mitochondrial IDH2, which produces NADPH, drives mitochondrial GSH turnover, and its downregulation increases sensitivity to erastin‐induced ferroptosis [91]. Interestingly, an IDH1 mutation in cholangiocarcinoma inhibits tumor progression by sensitizing cells to ferroptosis [92]. Mutant IDH1 produces the oncometabolite D‐2‐hydroxyglutarate (D‐2‐HG), responsible for the increase in ferroptosis sensitivity [93]. Inhibition of mutant IDH1 also confers resistance to erastin‐induced ferroptosis in the HT‐1080 fibrosarcoma cell line. IDH1/2 mutations are frequently observed in cancer entities such as acute myeloid leukemia, chondrosarcoma, cholangiocarcinoma, and glioblastoma.

Mechanistically, mutant IDH1 reduces GPX4 protein levels and promotes glutathione depletion, inducing ferroptosis [93]. Fumarate is an important intermediate in the TCA cycle and has been described to influence ferroptosis sensitivity in a certain setting. Dimethyl‐fumarate induces ferroptosis in diffuse large B‐cell lymphoma (DLBCL) by depleting GSH and inhibiting GPX4 [94]. Interestingly, inactivation of fumarate hydratase (FH) was found to promote ferroptosis sensitivity in renal cell cancer [95]. These data indicate that ferroptosis is a type of cell death closely intertwined with metabolic regulation, and the growing field of oncometabolism will likely identify additional metabolic vulnerabilities of cancer that can be targeted via the induction of ferroptosis.

## 2 The role of ferroptosis in cancer

### 2.1 Ferroptosis sensitivity, evasion, and escape in cancer

Cancer cells frequently present with increased levels of ROS, which can be caused by increased mitochondrial OXPHOS activity and ROS production but also by increased activity of ROS‐producing enzymes such as cyclooxygenases (COXs) and NADPH oxidases (NOXs). ROS act as crucial messenger molecules and play dual roles in cancer biology. On the one hand, high levels of ROS are frequently observed in cancer cells and are associated with cancer initiation and oncogenic transformation by transcriptional induction of proto‐oncogenes like Fos proto‐oncogene (c‐FOS), Jun proto‐oncogene (c‐JUN) and MYC proto‐oncogene, basic helix–loop–helix (bHLH) transcription factor (MYC) [96]. Many oncogenes, including HRAS, KRAS, and MYC, contribute to increased cellular ROS levels [97, 98, 99]. Furthermore, ROS released in inflamed tissues can be mutagenic, making tumors more malignant due to clonal diversification [100]. Increased ROS levels have been reported to promote proliferation and survival pathways, such as the phosphoinositide 3‐kinase (PI3K) pathway, via oxidative inactivation of phosphatase and tensin homolog (PTEN) and subsequent activation of AKT Serine/Threonine Kinase 1 (AKT) [101, 102]. On the other hand, studies describe that depletion of the ROS scavenger GSH and the consequent increase in ROS levels protect against tumor initiation but not tumor progression [103]. It is therefore likely that a delicate balance of ROS levels in tumors is crucial. This is supported by the finding that endogenous expression of oncogenic KRAS induces the antioxidant transcription factor NRF2, thereby reducing ROS levels and enhancing tumorigenesis [104]. Interestingly, the same study showed that exogenous expression of high levels of oncogenic KRAS rather suppressed activation of NRF2, which led to very high cellular ROS levels. Elevated oncogenic RAS expression has been recognized as a trigger for cellular senescence [105]. Notably, recent evidence demonstrates that fibroblasts expressing oncogenic KRASG12V exhibit protection against H_2_O_2_‐induced cell death by up‐regulating SLC7A11, thereby facilitating KRAS‐induced tumor growth *in vivo* [106]. Therefore, it is not surprising that recent findings hint at the pivotal role of ferroptosis in cancer cell biology [107, 108, 109, 110]. Intriguingly, ferroptosis has been implicated in the tumor‐suppressing function of p53 by suppressing SLC7A11 expression at the transcriptional level, sensitizing cells to ferroptosis [107]. Small cell lung cancer (SCLC) with bi‐allelic inactivation of *Trp53* and *Rb1* was shown to express elevated levels of SLC7A11, and the non‐neuroendocrine subset of SCLC was exquisitely sensitive to ferroptosis induction due to enhanced ether lipid synthesis [79]. Furthermore, p53 mutants, which have been found to accumulate in the cytosol, have been shown to bind and sequester NRF2, preventing the nuclear translocation of NRF2 and the induction of its target genes, including SLC7A11 [108]. Importantly, constitutive NRF2 activation resulting from a mutation of its negative regulator Kelch‐like ECH‐associated protein 1 (KEAP1) in non‐small cell lung cancer (NSCLC) was also shown to render cells resistant to ferroptosis due to the induction of FSP1 transcription [111]. Moreover, NRF2‐mediated transcription also drives up FSP1 expression in isogenic cells expressing oncogenic but not wild‐type KRAS [112]. Given the latter finding, it may not come as a surprise that deletion of GPX4 in a genetically engineered mouse model of PDAC was insufficient to induce tumor regression [113]. While systemic depletion or inhibition of SLC7A11 has shown promise as a therapeutic strategy in killing pancreatic and lung adenocarcinoma [114, 115], in pancreatic ductal adenocarcinoma (PDAC), this was attributable to cystine depletion in cancer‐associated fibroblasts (CAFs) rather than in PDAC cells [116]. This is consistent with the idea that KRAS‐mutated cells may require additional inhibition of FSP1 for efficient induction of ferroptosis [112]. In addition to oncogene‐induced ferroptosis rewiring, cells must pass through different metabolic tissue domains during metastasis. Interestingly, lymph‐borne metastasis was shown to be protected from ferroptosis due to increased levels of GSH and oleic acid within lymph fluid [81].

In contrast to the aforementioned studies in which p53 suppresses SLC7A11 expression, research on human colorectal cancer (CRC) cells revealed that p53 promotes SLC7A11 expression [117]. Moreover, the same study highlighted that the loss of p53 inhibits the accumulation of dipeptidyl‐peptidase‐4 (DPP4) in the nucleus, resulting in enhanced plasma‐membrane‐associated DPP4‐dependent lipid peroxidation via ROS‐generating NADPH oxidase enzymes, ultimately leading to ferroptosis [117]. Consequently, loss of p53 may sensitize cells to ferroptosis in specific contexts. Similar to the situation observed in SCLC, knockdown of the tumor suppressor gene retinoblastoma‐associated protein 1 (RB1) in hepatocellular carcinoma sensitizes cells to sorafenib‐induced ferroptosis [118]. Considering that components of the ferroptosis pathway are often aberrantly expressed in cancer cells [1, 2, 3], manipulation of the molecular mechanisms in the ferroptosis pathway might be exploited in cancer therapy. A particular field of interest has been founded with the discovery that drug‐tolerant persister cells (DTPs) and cells with mesenchymal transcriptional profiles acquire selective sensitivity to the induction of ferroptosis [109, 110]. These studies shed light on the possibility that resistance to targeted treatment might be tackled by ferroptosis‐inducing therapeutic regimes. However, while the main ferroptosis inducers (RSL3 and erastin) used *in vitro* have limitations for *in vivo* application [37, 119], efforts are underway to identify FDA‐approved drugs that induce ferroptosis in different cancer types, offering potential avenues for cancer therapy [48, 110, 120, 121].

### 2.2 Ferroptosis‐inducing agents (FINs) and sensitizers

Since the discovery of ferroptosis as a novel cell death modality, a lot of efforts have been focused on the development of specific small molecules that induce ferroptotic cell death. These small molecules can be classified into classes of ferroptosis‐inducing agents (FINs): class I FINs impair GPX4 activity by depleting its co‐factor GSH, e.g., erastin and imidazole ketone erastin (IKE) [121, 122]; class II FINs inhibit GPX4 directly, e.g., RSL3, ML162, and ML210; class III FINs include FIN56, which leads to GPX4 degradation [123]; and, lastly, class IV FINs act on iron oxidization such as FINO_2_ [124] (FINs are comprehensively reviewed elsewhere [125]). While FINs have been described based on the different points along the ferroptosis pathway they act on, their specific molecular targets and off‐targets are emerging from more recent studies.

GPX4 was initially identified as a binding target of RSL3 using an affinity pulldown assay and subsequent proteomics [37]. Eaton et al. [126] identified ML210 as another GPX4 inhibitor, hypothesizing that it binds to the selenocysteine residue of GPX4. Of note, RSL3 inhibits GPX4 by alkylating its selenocysteine moiety only upon the addition of cytosolic lysates, indicating that under physiological conditions, cytosolic adaptor proteins facilitate RSL3‐mediated inhibition of GPX4 [127]. Indeed, a recent study described that RSL3, ML210, and ML162 are not capable of inhibiting recombinant GPX4 in cell‐free systems but that RSL3 and ML162 are instead potent direct inhibitors of TXNRD1 [128]. Given that TXNRD1 is also a selenoprotein, the question arises whether RSL3 indeed has affinity towards other selenoproteins than TXNRD1 and GPX4. Erastin has also been shown to bind directly to mitochondrial voltage‐dependent anion channel 2 (VDAC2) using purified VDAC2 in a cell‐free affinity assay [2]. Erastin treatment and subsequent VDAC2 inhibition led to ROS production through a NADPH‐dependent pathway via mitochondrial damage [2]. FINs and their proposed mechanisms of action are summarized in Table 1. While FINs are under development, in some cases, targeting GPX4 is insufficient to effectively induce ferroptosis due to elevated expression of FSP1 [58, 59, 112]. Therefore, different FSP1 inhibitors have been developed. The first identified FSP1 inhibitor, iFSP1 [58, 59], is a human‐selective FSP1 inhibitor binding in the Quinone‐binding pocket, whereas recently identified viFSP1 [129] binds the NAD(P)H binding pocket in a species‐independent manner. The same group identified that icFSP1 inhibits FSP1 by different means, inducing condensation and the formation of droplet‐like structures of FSP1 [130]. Brequinar, a DHODH inhibitor, has been identified to sensitize cells to ferroptosis by, in fact, inhibiting FSP1 instead of DHODH [64]. Another recently identified molecule, FSEN1, sensitized to GPX4 inhibition, but the exact mechanism has not yet been described [131]. Taken together, while many ferroptosis‐inducing small molecules do not reach pharmacokinetic requirements for their effective clinical use yet, their optimization is a field of intense investigation and rapid development.

**Table 1:** List of available ferroptosis‐inducing agents (FINs).

	Name	Mechanism of action
FIN I	Erastin [1, 132]	System xc^−^ inhibitor
	Imidazole Ketone Erastin (IKE) [133]	System xc^−^ inhibitor
	Sulfasalazine [120, 134, 135, 136]	System xc^−^ inhibitor
	Sorafenib [48, 118, 121]	System xc^−^ inhibitor
	Cysteinase [137]	Cys depletion
	BSO [37, 103, 138]	GCL inhibitor
	Artesunate [139]	Glutathione S transferase
FIN II	RSL3 [22]	GPX4 inhibitor
	ML210 [37]	GPX4 inhibitor
	ML162 [37, 140]	GPX4 inhibitor
	Withaferin A [141, 142]	GPX4 inhibitor
	Altretamine [143, 144]	GPX4 inhibitor
FIN III	iFSP1 [110], icFPS1 [130], viFSP1 [129]	FSP1 inhibitor
	FSEN1 [60], Brequinar [64]	FSP1 inhibitor
	FIN56 [123, 145]	GPX4 degradation, squalene synthase inhibition
	Statins [110, 146, 147]	Blocks CoQ10 synthesis
FIN IV	Artemisinin [148]	Lysosomal degradation of ferritin & regulating the system xc^−^/GPX4 axis

## 3 The impact of ferroptosis on cancer‐associated inflammation

Cancer‐associated inflammation is a dynamic and important part of cancer as it fuels tumor initiation, modulates progression, and can ultimately promote metastasis. The tumor microenvironment (TME) contains immune cells comprised of T and B lymphocytes, tumor‐associated macrophages (TAMs), dendritic cells (DCs), natural killer (NK) cells, neutrophils, and myeloid‐derived suppressor cells (MDSCs). In addition, stromal cells such as CAFs, extracellular matrix (ECM) components, and other secreted molecules are in contact with tumor cells but also with each other [149].

### 3.1 Paracrine responses to ferroptotic cancer cells

Necrotic cell death programs are known to result in the release of danger‐associated molecular patterns (DAMPs) that can be recognized by pattern recognition receptors (PPRs), resulting in the activation of the immune system [150]. While proteomics from supernatants of cells undergoing necroptosis have revealed several bona fide inflammatory chemo‐ and cytokines to be present [151], unbiased approaches to catalogue ferroptotic supernatants are still lacking. Nevertheless, several classes of potential immune modulators released from ferroptotic cells have been studied. The release of oxidized phospholipids (oxPLs), the lipid peroxidation by‐product 4‐hydroxynonenal (4‐HNE), high‐mobility group protein B1 (HMGB1), and ATP were reported as potential immune modulators released from ferroptotic cells [152]. In a mouse model of PDAC, ferroptotic cells were proposed to release the oxidized nucleotide 8‐hydroxy‐2′‐deoxyguanosine (8‐OHdG) and activate the stimulator of interferon genes (STING) pathway, resulting in macrophage infiltration and pro‐tumor M2 polarization [113]. Ferroptotic cells have been shown to release HMGB1 in an autophagy dependent manner, leading to the activation of immune cells upon binding to the advanced glycosylation end‐product‐specific receptor (AGER) but not the Toll‐like receptor (TLR) 4 [153]. The kinetic release of exemplary DAMPs from ferroptotic cancer cells coupled to their inflammatory potential has also been reported in two studies comparing early and late ferroptotic cells [154, 155]. Both studies have shown the release of DAMPs such as HMGB1, ATP, and calreticulin on the surface of ferroptotic cancer cells. Efimova et al. [154] argue that ‘early’ ferroptotic cancer cells can be efficiently engulfed by bone marrow‐derived dendritic cells (BMDCs), stimulate their maturation and activation, and are able to protect mice from tumor development in a prophylactic vaccination manner. This, however, was not the case when using ‘late’ ferroptotic cells. By contrast, Wiernicki et al. [155] show that ‘early’ ferroptotic cells impede BMDC maturation and engulfment capacity, leading to decreased antigen cross‐presentation and dominant inhibition of an anti‐tumor immune response. Given that both studies make use of the same cell line model, it is likely that differences in the amount of cell death achieved by either RSL3 (in the case of Efimova et al.) or inducible knockdown (in the case of Wiernicki et al.) or metabolic differences might account for the discrepancies observed.

**Fig. 4:**
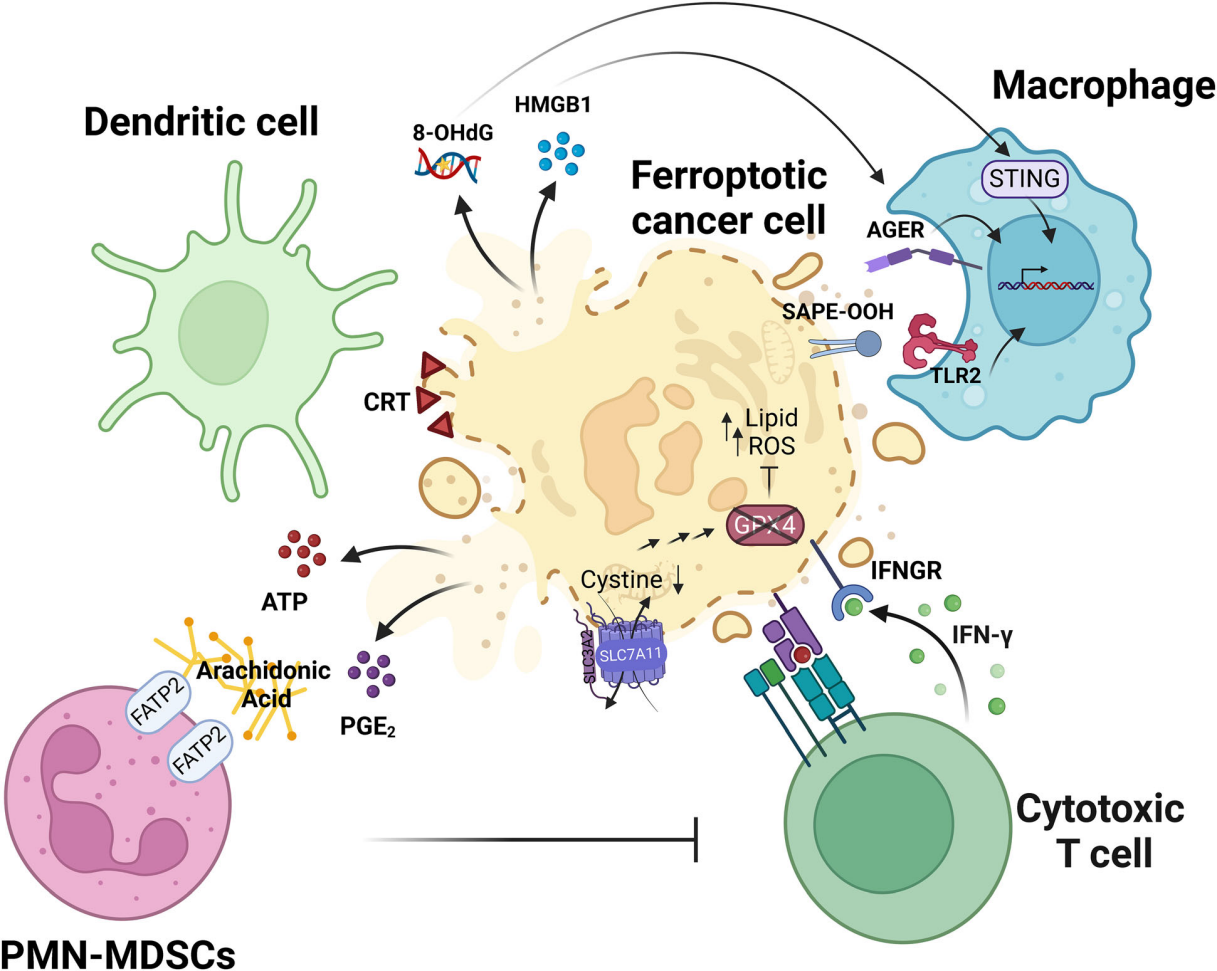
Immunomodulatory roles of ferroptosis in cancer. Ferroptosis induces the release of diverse classes of danger‐associated molecular patterns (DAMPs), resulting in the modulation of dendritic cell and macrophage responses. The oxidized phospholipid SAPE‐OOH can be recognized by TLR2 on macrophages to mediate phagocytosis. Moreover, released 8‐OHdG can bind to cGAS to activate STING‐mediated macrophage responses to support tumor progression. PMN‐MDSCs that inhibit the antitumor immune response can undergo ferroptosis, releasing immunosuppressive oxidized lipids to the tumor microenvironment (TME), affecting the promotion of tumor growth. The anti‐tumor immunity of CD8+ T cells can be modulated by ferroptosis. IFN‐γ secreted by activated CD8+ T cells can downregulate the expression of system X_c_^−^ (SLC7A11/SLC3A2), sensitizing tumor cells to ferroptosis. 8‐OHdG, 8‐Hydroxyguanosine; AGER, Advanced glycosylation end product‐specific receptor; ATP, Adenosine triphosphate; CRT, Calreticulin; FATP2, Fatty acid transport protein 2; GPX4, Glutathione peroxidase 4; HMGB1, High‐Mobility Group Box 1; IFNGR, Interferon‐gamma receptor; IFN‐γ, Interferon gamma; PGE_2_, Prostaglandin E2; PMN‐MDSCs, Polymorphonuclear myeloid‐derived suppressor cells; SAPE‐OOH, 1‐Stearoyl‐2′‐15‐HpETE‐sn‐glycero‐3phosphatidylethanolamine; SLC3A2, 4F2 cell‐surface antigen heavy chain; SLC7A11, Solute carrier family 7 member 11 (xCT); STING, Stimulator of interferon genes; TLR2, Toll‐like receptor 2. The figure has been created using Biorender.

In addition to the release of well‐established DAMPs, oxPLs can also be regarded as immunomodulatory [156]. For example, an oxidized phospholipid, 1‐steaoryl‐2‐15‐HpETE‐sn‐glycero‐3phosphatidylethanolamine (SAPE‐OOH), can act as an eat‐me signal on the surface of ferroptotic cells, which recruits macrophages via binding to TLR2 [157]. Oxidized phosphatidylcholine can inhibit the maturation and activation of BMDCs [158]. Increased levels of COX2, an enzyme that plays a vital role in generating the inflammatory mediator prostaglandin E2 (PGE2) from AA, have been identified as a distinctive feature of ferroptosis [37]. Indeed, knockdown of GPX4 in NIH‐3T3 cells resulted in the release of COX2‐dependent PGE_2_ and PGF_2α_ [159]. Elevated levels of PGE_2_ were also observed in keratinocytes derived from *Gpx4*‐deficient mice [160]. Taken together, ferroptotic cells can release a number of putative DAMPs and inflammatory mediators summarized in Fig. 4, yet an unbiased appraisal of these remains to be characterized.

### 3.2 Ferroptosis: pro‐ or anti‐tumor?

Given the fact that early on ferroptosis was recognized to constitute a lytic type of cell death, it was expected to lead to the release of DAMPs and, hence, to result in an anti‐tumor immune response. Confirming this assumption, the CD8 T‐cell‐mediated anti‐tumor attack raised through combined immune checkpoint blockade was shown to be reverted by co‐treatment with the ferroptosis‐selective RTA liproxstatin‐1 [161]. Moreover, CD8 T‐cell‐derived lFN‐γ secretion was shown to synergize with free AA to upregulate ACSL4‐mediated AA lipid integration and increase ferroptosis sensitivity in tumor cells [74] (Fig. 3). In contrast to these findings proposing a role for tissue ferroptosis in raising an anti‐tumor immune response, M2 macrophages were recently shown to be more sensitive to ferroptosis than M1 macrophages due to lower levels of nitric oxide synthase (iNOS) [162]. Unlike CD8 T‐cells, macrophages and neutrophil granulocytes are the first immune cells that are recruited to the site of inflammation [163]. TAMs are known to be highly plastic and can present with M1 or M2 phenotype, which manifest in tumor‐attack or tumor‐shielding activities, respectively. In keeping with the tumor‐shielding activity of tissue ferroptosis, pathologically activated neutrophils, termed myeloid‐derived suppressor cells (PMN‐MDSCs), were shown to die via ferroptosis. This caused the release of immunosuppressive oxygenated lipids via fatty acid transport protein *2* (FATP2), in turn leading to overall immunosuppression [164] (Fig. 4). Moreover, dendritic cells (DCs) exposed to ferroptotic cells were impaired in their capacity to cross present cancer‐associated antigens [155]. Of note, DCs impaired in cross presentation were shown to accumulate oxidatively truncated lipids from within lipid bodies, leading to intracellular trapping of MHC complexes [165, 166]. Further supporting ferroptosis‐induced immune suppression in cancer, the upregulation of the scavenger receptor CD36 on CD8+ T cells was shown to promote uptake of PUFAs, resulting in ferroptosis of CD8 T‐cells and impaired anti‐tumor immunity [167, 168]. Moreover, activated regulatory T cells (T_reg_) were sensitive to T_reg_‐selective *Gpx4* deletion, allowing for improved anti‐tumor immunity [169]. Taken together, these studies highlight that the effect of ferroptosis on various cell types within the TME is highly diverse. The data also propose that unlike other types of regulated necrosis, ferroptosis might elicit a type of immune modulation that seems to be highly dependent on the exact inflammatory context and, as such, ferroptotic cells might act more as an adjuvant than a *bona fide* inducer or inhibitor of inflammation.

## 4 Conclusions and perspectives

Ferroptosis is a mechanism of cell death that, owing to its unique mechanism, does not overlap with other cell death signaling pathways. Intracellular iron pools represent important prerequisites for many of the biochemical processes leading to lipid peroxidation. The currently known main arm of cellular ferroptosis protection revolves around GPX4 regulation of expression and activity through GSH synthesis, recovery, and localization to protect cells from detrimental lipid peroxidation. As a prerequisite for lipid peroxidation, the MUFA/PUFA ratio of cellular membranes is a strong determinant of ferroptosis sensitivity, with the synthesis of endogenous radical‐trapping agents acting as a potent protective strategy. With several indications that ferroptotic cells release several putative DAMPs and inflammatory mediators, ferroptosis‐based cancer therapy will likely involve modulators of an immune response. Currently, ferroptosis‐inducing small molecules do not reach pharmacokinetic requirements for their effective clinical use yet, but several efforts are under way to achieve clinical development. Importantly, sensitivity to ferroptosis is closely intertwined with the metabolic plasticity of cancer and is therefore anticipated to yield therapeutic successes in combating acquired resistance and in addressing treatment escape through plasticity.

## Conflict of interest

The authors declare no conflict of interest.

## Author contributions

FIY, CMB, and SK conceptualized the study. FIY and CMB conducted the literature review and generated the figures. SK reviewed and edited the study and acquired the funding. All authors have read and agreed to the published version of this manuscript.
